# Acceptance, Intent, Hesitance, and Attitudes Towards SAR-CoV-2 Vaccines Among Healthcare Workers in Michigan, USA

**DOI:** 10.7759/cureus.41225

**Published:** 2023-06-30

**Authors:** Utibe Effiong, Ifiok Umana, Henry Haley, Jocelyn Garcia, Chin-I Cheng, Neli Ragina, Frederick Eruo

**Affiliations:** 1 Internal Medicine, Central Michigan University (CMU) College of Medicine, Mt. Pleasant, USA; 2 Urology Division, Jos University Teaching Hospital, Jos, NGA; 3 Internal Medicine, MidMichigan Health, Midland, USA; 4 Statistics, Central Michigan University (CMU) College of Medicine, Mt. Pleasant, USA; 5 Medical Discipline, Central Michigan University (CMU) College of Medicine, Mt. Pleasant, USA; 6 Obstetrics and Gynecology, Northeast Ohio Medical University, Rootstown, USA

**Keywords:** covid-19, hesitance, vaccine, acceptance, healthcare workers, michigan, attitude

## Abstract

Background: Healthcare workers (HCWs) are critical infrastructure workers for whom COVID-19 vaccination was prioritized. It is believed that healthcare workers would have little or no hesitancy to take the COVID-19 vaccines given the risks of the pandemic to them, their families, and their patients.

Objective: The study aims to understand the acceptance and attitudes toward COVID-19 vaccines among the HCWs in Michigan.

Methods: A cross-sectional survey was fielded from January 11, 2021, through February 28, 2021. We obtained a representative sample of HCWs at MidMichigan Health. The participants were approximately 1500 clinical and non-clinical HCWs. COVID-19 vaccination acceptance and the intent to be vaccinated were measured with a questionnaire. HCWs indicating hesitance were asked to enter their reasons for hesitance as a free text response.

Results: A total of 1,467 HCWs responded to the survey. Overall, 62% indicated they had received both shots; 19.7% reported that they had received the first shot and would take the second; 2.3% noted that they were yet to receive the vaccine but would take both shots; 0.4% reported that they had received the first shot but would not take the second; 5.7% noted that they were unsure; and 9.9% indicated they did not intend to take the vaccine. Factors associated with vaccine hesitance included being female, younger age, having administrative staff or other health workers, having a larger household size, and having received no vaccines in the past year. Vaccine hesitancy concerns included safety, efficacy, antivaccine beliefs, the need for additional information, and a lack of trust.

Conclusion: This survey revealed that 16% of HCWs in central and northern Michigan were hesitant about COVID-19 vaccines. Vaccine education is needed to increase the acceptance of COVID-19 vaccines among HCWs.

## Introduction

In late December 2019, an investigation of a cluster of pneumonia cases of unknown origin in Wuhan, China, resulted in the identification of a novel coronavirus. The virus, later named severe acute respiratory syndrome coronavirus 2 (SARS-CoV-2), was distinct from both the 2002-2004 severe acute respiratory syndrome coronavirus (SARS-CoV-1) and Middle East respiratory syndrome coronavirus (MERS-CoV), although closely related [1]. The World Health Organization (WHO) announced "COVID-19" as the name of this new disease on February 11, 2020, following guidelines previously developed with the World Organization for Animal Health and the Food and Agriculture Organization of the United Nations [2].

COVID-19 has become a significant public health crisis, affecting more than 162.1 million individuals and causing over 3.3 million deaths globally by May 2021. The United States (US) has reported more than 32 million cases and 585,407 deaths as of May 15, 2021 [3]. To curb this pandemic, effective public health measures such as social distancing, wearing face masks, hand washing, avoiding crowded indoor spaces, educating the general population, and vaccination have emerged as essential to mitigating disease and death [4].

A report by WHO in 2020 showed that most deaths from COVID were seen in people who were not vaccinated; this was a glaring demonstration that the vaccines were actually effective [5]. This validated the first emergency authorization by the Food and Drug Administration (FDA) for the COVID-19 vaccines. Following this authorization, priority for getting the shot was given to risky groups of populations like healthcare workers (HCWs) and teachers [6].

However, the role of the media in propagating vaccine hesitancy cannot be overlooked. Wilson and Wiysonge in 2020 [7] showed the integral role played by the media in causing vaccine hesitancy through the use of anti-vaccine propaganda.

Clinicians and other healthcare providers play a crucial role in parental decision-making about immunization. Healthcare providers are cited by parents, including parents of unvaccinated children, as the most frequent source of information about vaccination [8]. HCWs have also been identified as critical infrastructure workers for whom early SAR-CoV-2 (COVID-19) vaccination has been prioritized and offered [9,10]. Therefore, healthcare leaders and the public may assume that HCWs would have little hesitancy to take the COVID-19 vaccination given the risks of the pandemic to them, their families, and their patients [9]. As such, it is crucial to consider HCW attitudes about COVID-19 vaccination to better address barriers to widespread vaccination [9].

## Materials and methods

Participants and survey administration

To understand general vaccination acceptance and specific attitudes toward coronavirus vaccines among the HCWs in the central and northern regions of the state of Michigan, we conducted a cross-sectional survey of HCWs employed by or affiliated with MidMichigan Health. MidMichigan Health is an extensive health system affiliated with the University of Michigan Health System (now known as Michigan Medicine). This health system serves central and northern Michigan. About 8,000 HCWs are either employed by or affiliated with MidMichigan Health. The health system began offering two-dose COVID-19 vaccines in December 2020.

We surveyed a representative sample of HCWs employed by or affiliated with MidMichigan Health using a validated self-administered online instrument. The survey questionnaire was created on the Qualtrics XM platform, licensed to the University of Michigan. A link to the survey was emailed to all employees and affiliates of MidMichigan Health. Weekly reminders for survey completion were also emailed. The survey was conducted from January 11, 2021, through February 28, 2021.

Our study was determined to be exempt by the MidMichigan Health Institutional Review Board.

Measures

The demographic information collected included gender, age, race, profession, marital status, household size, and household setting. The general attitude towards vaccination was accessed with the question, "Have you received any vaccinations in the past year?" Table 1 summarizes the demographic information obtained. We categorized HCWs as physicians (MD or DO), advanced practice providers (NP or PA), nurses, administrative staff, or other health workers.

**Table 1:** Descriptive statistics in count (percentage) for the variables

Variable	Total (n=1,467)
Gender	
Male	164 (11.18%)
Female	1302 (88.75)
Other	1 (0.07%)
Age	
18–29	165 (11.25%)
30–44	491 (33.47%)
45–64	757 (51.60%)
65–74	52 (3.54%)
75 and above	2 (0.14%)
Race	
Asian, non-Hispanic	10 (0.68%)
Black, non-Hispanic	7 (0.48%)
Hispanic	19 (1.30%)
White, non-Hispanic	1415 (96.46%)
Others	16 (1.09%)
Profession	
Physician (MD, DO)	39 (2.66%)
Advanced practice provider (NP, PA)	33 (2.25%)
Nurse	402 (27.40%)
Administrative staff	326 (22.22%)
Other health workers	667 (45.47%)
Marital status	
Married	1039 (70.82%)
Living with partner	103 (7.02%)
Widowed	23 (1.57%)
Divorced or separated	150 (10.22%)
Never married	152 (10.36%)
Household size	
1	147 (10.02%)
2	541 (36.88%)
3	290 (19.77%)
4	300 (20.45%)
5	138 (9.41%)
6 or more	51 (3.48%)
Household setting	
Urban	496 (33.81%)
Rural	971 (66.19%)

COVID-19 vaccination acceptance and the intent to be vaccinated were measured with the question, "What is your COVID-19 vaccine completion status?" Response options were: "I have received both shots," "I have received the first shot, and I will take the second shot," "I am yet to receive the vaccine, but I will take both shots," "I have received the first shot, but I will not take the second shot," "I am yet to receive the vaccine, and I am still unsure," and "I do not intend to take the vaccine."

Hesitance and attitudes towards COVID-19 vaccines were further assessed with the instruction, "If you are unsure or do not intend to take one or both shots of the vaccine, please provide a reason for your response." Participants entered free-text responses to this instruction.

Statistical analysis

Statistical analysis was primarily conducted using SAS software, version 9.4 (SAS Institute, Inc., Cary, NC). Descriptive statistics of count and percentage were provided for categorical variables. A multivariable logistic regression model was adopted to identify associations between attitudes toward the COVID-19 vaccine and demographic variables. The Hosmer-Lemeshow statistic was not statistically significant (p-value = 0.77800), indicating excellent model fit and performance. With all the variance inflation factors being less than 10, the proposed model did not have multicollinearity issues. The 95% confidence interval for the odds ratio, p-values, and upper and lower limits were listed. All of the analytical results were considered significant when p-values were less than or equal to 0.05.

## Results

The MidMichigan Health COVID-19 Vaccine Survey was released to a total of 7,784 HCWs employed by or affiliated with the health system. The survey was completed by 1,467 participants, representing 18.8% of the target population. Table 1 summarizes the descriptive statistics for this survey.

A majority (88.8%) of the survey respondents were female. One respondent (0.1%) identified as "other," while 11.2% identified as males. The age distribution of the respondents was as follows: 11.3% were aged 18-29, 33.5% were 30-44, 51.5% were 45-64, 3.5% were 65-74, and only 0.1% were aged 75 and above. Virtually all the respondents (96.5%) were non-Hispanic White. Non-Hispanic Asians made up 0.7% of respondents; 0.5% were non-Hispanic Black; 1.3% were Hispanic; and 1.1% identified as "other" races.

Most (45.5%) respondents also identified as "other health workers." Nurses were 27.4%, administrative staff were 22.2%, physicians were 2.7%, and advanced practice providers (APPs) - nurse practitioners (NPs) and physician assistants (PAs) - were 2.3% of the respondents. Most (70.8%) were married. Divorced or separated respondents were 10.2%, 10.4% indicated they were never married, 7% lived with a partner, and 1.6% were widowed.

Regarding the respondents' household sizes, 10% had only one member, 36.8% had two, 19.8% had three, 20.5% had four, 9.4% had five, and 3.5% had six or more members. Most (66.2%) of respondents lived in a rural setting, while 33.8% lived in an urban area.

A majority (88%) of respondents indicated that they had received a vaccine within the last year, while 12% had not. A majority (62%) of participants indicated that they had completed their COVID vaccination series. Respondents who had received the first shot and intended to take the second shot made up 19.7% of the study population, while 2.3% reported that they had yet to receive the vaccine but intended to take both shots.

Nearly a tenth (9.9%) of the study population indicated that they did not intend to take the COVID-19 vaccine; 0.4% reported taking the first shot but noted that they would not take the second; and 5.7% indicated that they were yet to receive the vaccine and noted that they were still unsure.

Overall, 84% of participants were acceptant ("yes") of the COVID-19 vaccine, 5.7% were unsure ("not sure"), and 10.3% were not acceptant ("no") of the vaccine. The acceptance, intent, and hesitance rates within our study population are depicted in Table 2.

**Table 2:** The acceptance, intent, and hesitance rates

Past vaccine	
Yes	1291 (88.00%)
No	176 (12.00%)
COVID vaccine	
I have received the first shot and I will take the second shot	289 (19.70%)
I am yet to receive the vaccine but I will take both shots	34 (2.32%)
I have received the first shot BUT I WILL NOT TAKE THE SECOND SHOT	6 (0.41%)
I am yet to receive the vaccine and I AM STILL UNSURE	83 (5.66%)
I DO NOT INTEND TO TAKE THE VACCINE	145 (9.88%)
I have received both shots	910 (62.03%)

We conducted a multivariable logistic regression (Tables 3-4) to examine the association between the COVID vaccine and demographic variables. For this analysis, we combined the "not sure" and "no" categories into a single category, "NO," to evaluate the odds of vaccine acceptance ("YES") and hesitance ("NO"). Factors associated with vaccine acceptance included male gender, middle-aged (age group 45-64), clinical staff status (physicians, APPs, and nurses), smaller household size, and having received any vaccine in the past year. Factors associated with vaccine hesitance included females, younger age groups, administrative staff and other health workers, larger household sizes, and not having received any vaccines in the past year.

**Table 3:** Multivariable logistics regression examined the association between the COVID vaccine and demographic variables, respectively. Odds ratios, p-value, and 95% confidence Intervals were listed.

	COVID vaccine yes vs no			
	OR	95% for OR		P-value
Variables		Lower	Upper	
Male vs Female	2.275	1.227	4.216	0.0090
Age 18–29 vs 30–44	0.693	0.405	1.185	0.1802
Age 18–29 vs 45–64	0.313	0.183	0.535	<0.0001
Age 18–29 vs 65–74	0.453	0.154	1.337	0.1516
Age 30–44 vs 45–64	0.452	0.310	0.659	<0.0001
Age 30–44 vs 65–74	0.654	0.235	1.819	0.4163
Age 45–64 vs 65–74	1.449	0.533	3.939	0.4677
White vs non-White	1.194	0.522	2.727	0.6748
Nurse vs MD, DO, NP, PA	0.313	0.090	1.089	0.0679
Administrative staff vs MD, DO, NP, PA	0.196	0.056	0.680	0.0103
Other health workers vs MD, DO, NP, PA	0.273	0.080	0.932	0.0383
Nurse vs administrative staff	1.596	1.033	2.467	0.0351
Nurse vs other health workers	1.148	0.778	1.693	0.4878
Administrative staff vs other health workers	0.719	0.488	1.059	0.0948
Widowed/divorced/separated vs married/living with partner	0.843	0.452	1.574	0.5926
Never married vs married/living with a partner	1.519	0.790	2.922	0.2104
Widowed/divorced/separated vs never married	0.555	0.239	1.290	0.1712
Household size 1 vs 2	4.513	1.436	14.183	0.0099
Household size 1 vs 3	6.918	2.170	22.057	0.0011
Household size 1 vs 4	7.402	2.277	24.063	0.0009
Household size 1 vs 5 or more	9.268	2.784	30.856	0.0003
Household size 2 vs 3	1.533	0.976	2.407	0.0636
Household size 2 vs 4	1.640	1.042	2.582	0.0327
Household size 2 vs 5 or more	2.053	1.242	3.394	0.0050
Household size 3 vs 4	1.070	0.673	1.701	0.7748
Household size 3 vs 5 or more	1.340	0.805	2.231	0.2610
Household size 4 vs 5 or more	1.252	0.773	2.029	0.3616
Rural vs urban	0.725	0.509	1.034	0.0761
Past vaccine yes vs no	10.331	7.095	15.044	<0.0001

**Table 4:** Multivariable logistics regression examined the association between the COVID vaccine and demographic variables, respectively. Odds ratios, p-value, and 95% confidence intervals were listed.

	COVID Vaccine no vs yes			
	OR	95% for OR		P-value
Variables		Lower	Upper	
Male vs female	0.440	0.237	0.815	0.0090
Age 18–29 vs 30–44	1.444	0.844	2.471	0.1802
Age 18–29 vs 45–64	3.196	1.87	5.464	<0.0001
Age 18–29 vs 65–74	2.206	0.748	6.509	0.1516
Age 30–44 vs 45–64	2.214	1.518	3.229	<0.0001
Age 30–44 vs 65–74	1.528	0.55	4.249	0.4163
Age 45–64 vs 65–74	0.69	0.254	1.877	0.4677
White vs non-White	0.838	0.367	1.915	0.6748
Nurse vs MD, DO, NP, PA	3.195	0.918	11.124	0.0679
Administrative staff vs MD, DO, NP, PA	5.101	1.47	17.706	0.0103
Other health workers vs MD, DO, NP, PA	3.667	1.073	12.538	0.0383
Nurse vs administrative staff	0.626	0.405	0.968	0.0351
Nurse vs other health workers	0.871	0.591	1.286	0.4878
Administrative staff vs other health workers	1.391	0.944	2.049	0.0948
Widowed/divorced/separated vs married/living with partner	1.186	0.635	2.214	0.5926
Never married vs married/living with a partner	0.658	0.342	1.266	0.2104
Widowed/divorced/separated vs never married	1.801	0.775	4.184	0.1712
Household size 1 vs 2	0.222	0.071	0.696	0.0099
Household size 1 vs 3	0.145	0.045	0.461	0.0011
Household size 1 vs 4	0.135	0.042	0.439	0.0009
Household size 1 vs 5 or more	0.108	0.032	0.359	0.0003
Household size 2 vs 3	0.652	0.415	1.024	0.0636
Household size 2 vs 4	0.61	0.387	0.96	0.0327
Household size 2 vs 5 or more	0.487	0.295	0.805	0.0050
Household size 3 vs 4	0.935	0.588	1.486	0.7748
Household size 3 vs 5 or more	0.746	0.448	1.243	0.2610
Household size 4 vs 5 or more	0.799	0.493	1.294	0.3616
Rural vs urban	1.378	0.967	1.965	0.0761
Past vaccine yes vs no	0.097	0.066	0.141	<0.0001

The odds of receiving the COVID-19 vaccine were 127.5% higher for male participants than for female participants (OR 2.275, 95% CI, 1.227-4.216). Middle-aged respondents were also more likely than younger participants to accept the vaccine; compared to those aged 18-29, the OR was 0.313 (95% CI, 0.183-0.535), and compared to those aged 30-44, the OR was 0.452 (95% CI, 0.310-0.659). The odds of vaccine acceptance for physicians or AAPs were 410.2% higher than those for administrative staff participants, and they were 266.3% higher than those for participants who were "other health workers." Nurses also had higher odds of vaccine acceptance than administrative staff (OR 1.596 [95% CI, 1.033-2.467]). Respondents living alone also had higher odds of vaccine acceptance when compared to those living with one or more people. Those living in a two-person household also had significantly higher odds of vaccine acceptance when compared to those in households of four or more persons; the OR was 1.640 (95% CI, 1.042-2.582) and 2.053 (95% CI, 1.242-3.394) for two versus four-person and five or more-person households, respectively. The odds of COVID-19 vaccine acceptance for participants who received any vaccine in the past year were 933.1% higher than those for participants who did not. These results are detailed in Tables 3-4.

Of the 234 respondents who indicated that they were unsure or did not intend to take one or both shots of the vaccine (hesitant participants), 190 (81.2%) provided a reason for their response. Table 5 summarizes the reported reasons for COVID-19 vaccine hesitance among the respondents. Most (54.7%) of the hesitant participants reported specific concerns regarding vaccine safety and efficacy; 12.1% noted that they required additional information; 15.8% indicated antivaccine attitudes, beliefs, and emotions; and 2.6% reported a lack of trust in the government and the CDC. The rest of the hesitant participants (14.7%) reported other reasons, including altruism (wanting higher-risk individuals to get the vaccine first), being previously positive for COVID-19, and breastfeeding or pregnancy.

**Table 5:** Reasons participants provided for responding “unsure” or “do not intend to take one or both shots of the vaccine”

Specific concerns about the vaccine	98 (51.6%)
Side effects, safety	55 (28.9%)
Efficacy	7 (3.7%)
Newness, including not wanting to be the first to get the vaccine	17 (8.9%)
The rigor of testing/speed of development	18 (9.5%)
Vaccine contents	1 (0.5%)
Need additional information	23 (12.1%)
Compatibility with personal health conditions (e.g. allergies, comorbidities)	16 (8.4%)
Need more information, unspecified	7 (3.7%)
Antivaccine attitudes, beliefs, and emotions	30 (15.8)
Don’t need the vaccine (e.g. not at risk)	9 (4.7%)
Religious beliefs	5 (2.6%)
Don’t believe the vaccine will work, informed by reference to other bad vaccines experiences, flu shot, not working, the vaccine won’t work against mutating organism	4 (2.1%)
General statements about not getting the vaccines	6 (3.2%)
Don’t believe in, want, or feel comfortable with vaccines	1 (0.5%)
Mention mask requirements after the vaccine	4 (2.1%)
Misconceptions, or incorrect information about vaccines	1 (0.5%)
Lack of trust	5 (2.6%)
Government and the CDC	5 (2.6%)
Other	34 (17.9%)
Altruism, wanting higher-risk individuals to get the vaccine first	3 (1.6%)
Previously positive for COVID	11 (5.8%)
Reactions to the first dose	3 (1.6%)
Have seen others have reactions to the vaccine	3 (1.6%)
Breastfeeding/pregnancy	14 (7.4%)

## Discussion

In this system-wide survey of healthcare workers at MidMichigan Health, 16% of participants indicated some form of hesitance toward the COVID-19 vaccine. This vaccine hesitancy rate is much lower than the national rate of 42% that Fisher et al. [11] reported. However, it is still important to explore the causes of vaccine hesitancy among healthcare workers. Healthcare workers have some of the highest SARS-CoV-2 exposure rates, so we expect that a significant proportion of this population would be willing to be vaccinated. Healthcare workers are also leaders in our communities. Therefore, motives for vaccine hesitancy must be thoroughly assessed in this population. If vaccine hesitancy is not addressed in this population, misinformation or misconceptions regarding COVID-19 vaccination could prevent adequate COVID-19 vaccination in the general public.

The COVID infodemic, which is an overabundance of information, some of which is partly true and others partly false, played a huge role in the increased incidence of vaccine hesitancy in the US [12]. Since healthcare workers play critical roles in communities, they can influence vaccine acceptance to a greater extent. In a recent study, participants indicated that they were most likely to accept the vaccine if a healthcare professional recommended it [13].

It is well known that the COVID-19 vaccination is necessary to reduce COVID-19-related hospitalizations, the strain on hospital capacity, and deaths [11]. While at work, healthcare workers are directly affected by increased hospital occupancy and patient mortality. Sanghera et al., in their study in 2020, demonstrated worse mental health outcomes in healthcare workers with direct exposure to SARS-CoV-2 patients [14,15]. Regardless of the COVID-19 vaccination implications for their mental health, 145 out of 1467 healthcare workers (9.8%) in our study did not intend to take the vaccine. At the time of the survey, another 83 healthcare workers (5.7%) were still unsure if they wanted to receive the COVID-19 vaccine.

More than half of healthcare workers (51.6%) who displayed some vaccine hesitancy reported a specific concern about the vaccine. These included the rigor of testing or speed of development (9.5%), the newness of the vaccine (8.0%), the side effect/safety profile (28.9%), and efficacy (3.7%), which were the most significant reasons for vaccine hesitancy in this group. Fisher et al. uncovered a similar specific concern for the vaccine in the general public; 57% of the United States population surveyed that responded "no" or "not sure" noted the rigor of testing (3.9%), the newness of the vaccine (14.5%), efficacy (14.9%), and side effect/safety profile (29.4%) as their reasons for vaccine hesitancy.

Comparing our study with Fisher et al., a reduction of 6.5% was seen in both concerns for the rigor of testing and newness, including not wanting to be the first to get the vaccine. A reduction of 11.2% was seen between our study and the study by Fisher et al. regarding vaccine efficacy [11]. These differences could be due to healthcare workers' knowledge of how vaccines are brought to market, how they work, and their proven efficacy in fighting other highly virulent pathogens. Alternatively, this difference could be due to the timing of our studies. Fisher et al. conducted their survey in 2020, before a vaccine was available and before Pfizer released the first data on COVID-19 vaccination efficacy. Regardless of this difference, specific concerns about the vaccine are still the main reason behind vaccine hesitancy among healthcare workers and the general public.

The following was the most notable reason for vaccine hesitancy in our study: compatibility with personal health conditions. Of the 190 respondents who gave a reason for their vaccine hesitance, 16 (8.4%) cited a personal health condition or allergy as their reason for COVID-19 vaccination hesitancy. This is an interesting point of conversation because there have been multiple anecdotal reports of anaphylactic reactions following the COVID-19 vaccination. There was enough concern that the CDC changed its guidelines and now requires patients to be monitored for 15 minutes after vaccination or 30 minutes if they have a history of anaphylaxis or an immediate allergic reaction of any severity to a vaccine or injectable therapy [15].

Moreover, in 2021, the Journal of the American Medical Association (JAMA) published an article that addressed acute allergic reactions to mRNA COVID-19 vaccines [16]. Blumenthal et al. reported in 2021 that "anaphylaxis to the mRNA COVID-19 vaccines is currently estimated to occur in 2.5 to 11.1 cases per million doses, largely in individuals with a history of allergy" [16]. Hence, anaphylaxis occurs in much less than 1% of all vaccinations.

Furthermore, to our knowledge, there is no data to support a severe risk associated with vaccinating individuals with underlying health conditions. Besides, no currently available vaccine is a live attenuated virus; thus, immunocompromised individuals can be safely vaccinated and protected against SARS-CoV-2 [17].

Another important reason cited for vaccine hesitancy in our study was breastfeeding, pregnancy, and fertility concerns. Of the hesitant population in this study, 14 respondents cited concerns about breastfeeding, current pregnancy, or future fertility. Many survey respondents also indicated concern for the lack of evidence or differing opinions between the CDC, specialty medical groups, the FDA, and other medical professionals regarding pregnancy and breastfeeding. Their concern about the lack of evidence did have some merit. At the time of our survey, these vaccines were still fresh on the market and had not been thoroughly studied in specific populations, such as pregnant or breastfeeding women. However, there are no scientific reasons to believe COVID-19 vaccinations would adversely affect breastfeeding, current pregnancy, or future fertility. Nearly all routine vaccines are permitted during pregnancy except for live-attenuated vaccines, which are contraindicated because of the theoretical risk of the virus crossing the placenta and infecting the fetus. At the time of this survey, the two available authorized COVID-19 vaccines use messenger RNA (mRNA) technology. After injection, the unstable mRNA is quickly degraded inside tissue cells, with a minimal theoretical chance of crossing the placenta. Furthermore, the CDC, the American College of Obstetricians and Gynecologists (ACOG), and the Society for Maternal-Fetal Medicine (SMFM) state that pregnant individuals who meet the criteria for receiving the COVID-19 vaccine may choose to be vaccinated [18].

Although it is essential to analyze why healthcare workers may be hesitant to be vaccinated against COVID-19, it is also imperative to look at measurable underlying factors that may lead to vaccine hesitancy. Our study found that women were 4.1 times more likely to be hesitant about receiving the COVID-19 vaccine than their male counterparts. One explanation for this could be the previously discussed perceived risk a woman puts on their baby if breastfeeding, their unborn child if pregnant, or trying to become pregnant. To the best of our knowledge, there has been no data to support these claims. However, at the time of this study, there was not much research on this population of patients.

Our study also shows a linear relationship between household size and acceptance of the COVID-19 vaccine.

There could be several reasons for this trend; one we propose is that survey participants with a household size of one were less likely to be worried about pregnancy, breastfeeding, or future fertility, and consequently less worried about alleged COVID-19 vaccine complications related to pregnancy.

Survey participants 18 to 29 years of age were also 3.2 times more likely to display hesitation toward the COVID-19 vaccine than participants between the ages of 45 and 64. This finding could also be associated with worries about pregnancy, breastfeeding, or future fertility in the reproductive age group.

Another underlying characteristic of those with vaccine hesitancy is their role in healthcare. Our study found that administrative staff were 5.1 times more likely to be vaccine-hesitant than physicians or advanced practice providers and 1.6 times more likely than nursing staff. The difference seen among healthcare professionals may be due to a perceived risk of SARS-CoV-2 infection, or lack thereof. Understandably, administrative staff may have less overall occupational exposure to SARS-CoV-2. However, this is not true for all administrative staff, as some of them, such as registration officers and hospital ward clerks, do come into close contact with infectious patients.

The last point to discuss was the most significant data point of our study. Participants who had not had a vaccine in the past year were 10.3 times more likely to be hesitant toward the COVID-19 vaccine. We found that 40.2% of survey participants who displayed hesitancy against the COVID-19 vaccine did not receive a routine vaccine within the past year, compared to 93.4% of survey participants with no hesitancy against the COVID-19 vaccine who have received a routine vaccine in the past year. All healthcare workers at MidMichigan Health are required to receive the influenza vaccine. If they choose not to be vaccinated, they must go through a thorough process to opt out. There are many reasons these healthcare workers could have opted out of this routine vaccine. However, we propose that this proportion of our study population may demonstrate vaccine hesitancy toward all vaccines. This is the population that we need to educate the most. However, this population may be the most difficult to counsel on the topic of vaccination.

The strength of our studies lies in the large number of respondents and the sizable proportion that indicated their reasons for COVID-19 vaccine hesitance. In addition, the timing of the survey coincided with the inception of COVID-19 vaccinations in the United States, making our findings regarding healthcare workers, particularly salient.

A directive counseling approach can be utilized as one of the ways to address the hesitancy of COVID-19 vaccination by healthcare workers. Additionally, deliberate attempts at promoting the communication of proper and accurate information across social media channels can be effective in increasing vaccine uptake [19].

This study had several notable limitations. For one, our survey relied upon self-reporting of participants' roles in healthcare. Categories such as "administrative staff" and "other healthcare workers" are broad and not well defined. Future studies may include a breakdown of administrative and other healthcare roles. Also, the data were skewed towards respondents who reported themselves as either female or other health workers. Further, participants' acceptance and hesitance toward the COVID-19 vaccines were assessed during the first three months after vaccination commenced in the United States. As more details regarding the vaccines are known, some participants may change their responses on future surveys.

## Conclusions

In conclusion, we found that 84% of healthcare workers in central and northern Michigan were accepting of the COVID-19 vaccines, and 16% indicated hesitance. We determined that healthcare workers and the general public have similar qualms regarding the COVID-19 vaccines. The most notable were specific concerns about the vaccine, such as side effects, safety, and the newness of the vaccines. Compatibility with personal health conditions and concerns related to breastfeeding and pregnancy were also common causes of apprehension. Our study also determined that gender, household size, role in healthcare, and past-year vaccination status played a significant role in COVID-19 vaccine acceptance. We hope that other healthcare systems can use our data to educate their healthcare workers and increase the vaccination rate in their communities.
